# ‘Community reporting’: an insight-generating approach for local authority physical activity provision

**DOI:** 10.1177/17579139221102232

**Published:** 2022-07-14

**Authors:** AJ Potts, J McKenna, C Webber

**Affiliations:** Institute for Sport, Physical Activity, and Leisure, Leeds Beckett University, Leeds LS6 3QS, UK; Institute for Sport, Physical Activity, and Leisure, Leeds Beckett University, Leeds, UK; Institute for Sport, Physical Activity, and Leisure, Leeds Beckett University, Leeds, UK

## Introduction

Research into physical activity (PA) promotion often takes a top-down approach, meaning that it overlooks the experiences of local people.^1^ Recently research has acknowledged the importance of community-informed research as critical for understanding local contexts and for exploring health disparities and inequalities.^2^ Community insights are important for shedding light on how intrapersonal factors (e.g. self-concept), dynamic interpersonal relationships (e.g. friends, colleagues) and the local environment (e.g. parks and green spaces, workplaces) can influence PA both independently and in combination with other factors.^3^ However, community insights are often elusive using traditional research methods which typically involve interviews^4^ or focus groups.^5^ The potential of such methods is often undermined by local people being guarded about discussing personal and/or sensitive information with someone outside of their community.^6^

Previous literature highlights the challenges facing ‘out-group’ researchers – individuals regarded as ‘different’ due to their education, research expertise, race and/or socioeconomic status that may denote a more elevated privilege and power within society.^7^ While ‘out-group’ researchers may be objective and emotionally distant from the research process, they may find it difficult to gain access to research participants.^8^ ‘Out-group’ researchers may lack underpinning local knowledge, which often reduces empathy and the potential for research participants to experience the psychological safety needed to disclose their experiences.^9^

In light of these potential shortcomings, this article presents a novel approach to gaining community insight called ‘community reporting’ (CR). CR can provide an opportunity to engage with local residents who may otherwise be reluctant to share their experiences with ‘outsiders’. It is essential these experiences are captured to help develop case study examples to inform policy recommendations and action when creating healthy environments. This approach can go beyond being just ‘practical examples’ and instead influence decision making and, by using local context, can help to convince decision makers.^10^

**Figure:**
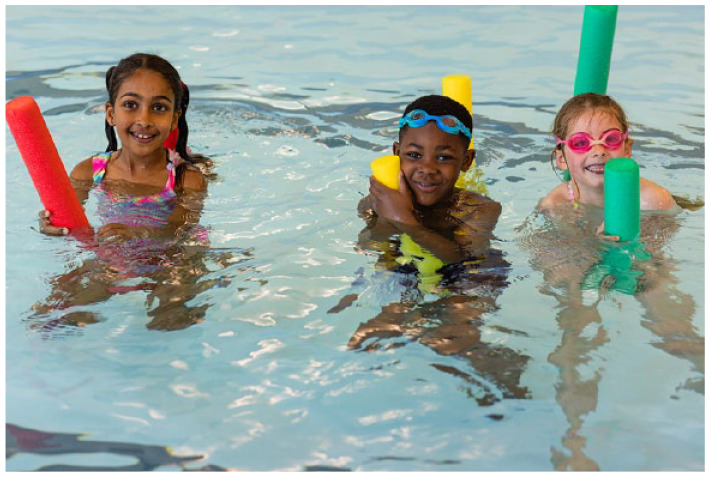


## In Practice

### Case study: Active Calderdale

Drawing on the insight-gathering work of the Sport England funded Local Delivery Pilot (LDP) ‘Active Calderdale’, which is using a whole-systems approach to PA promotion across the Borough, CR was identified as a functional and sensitive approach. CR was piloted in one locality to develop an understanding of the key organisations and services that were influential in directing PA behaviour. To maximise learning, the CR approach was one of a number of innovative approaches used within the larger evaluation and insight work of Active Calderdale. This process was instigated and delivered by an embedded researcher (AP) within Active Calderdale.

### Identifying community reporters

Following institutional ethical approval, community reporters were recruited through a Community Engagement Coordinator (CEC) who works for a local community anchor organisation partnered with Active Calderdale. Using their local knowledge, the CEC identified residents who were not only actively involved with community-based initiatives but also well connected to residents with limited social networks. These residents were approached individually to engage in the task.

### Workshops to train community reporters

A workshop was used to train the Community reporters, which took a four-step approach to the training:

1. Introduction (30 min)
The Community reporters were briefed on Active Calderdale and the insight-gathering task. This involved presenting the aims of Active Calderdale, the aims of the insight-gathering task and the proposed approach. The Community reporters had time to discuss Active Calderdale and ask any pertinent questions (e.g. how will the information gathered from this task be used?); it was important they fully understood the strategy and the task before proceeding.
2. Training and ethical considerations (30 min)
Next, AP familiarised the Community reporters with the conversation brief to be used with residents. It was important that these conversations were unstructured and followed the flow of conversation, rather than following a set agenda. They were encouraged to revert to the brief when conversation was beginning to tire. For example, topics pertinent to this project are related to (1) daily, weekly and monthly contacts to understand key influencers (e.g. can you tell me about who you speak to on a daily basis in the community?), (2) methods of travel in the area (e.g. can you tell me how you get to your local shop?) and (3) weekly work and/or leisure schedules (e.g. can you talk me through what your working week looks like?). To illustrate how the conversation might progress, AP and the CEC engaged in a role-play task. The Community reporters were also made aware of key ethical procedures that required adherence, such as confidentiality, the process of gaining consent and information about the location of each conversation.
3. Practice (45 min)
An essential part of the workshop was ensuring the opportunity to become fluent using the conversation brief. Community reporters took turns using the brief with fellow Community reporters, receiving constructive feedback from AP, the CEC and the other Community reporters in the group. Feedback typically revolved around how to initiate (e.g. can you tell me about local community groups you engage with?), develop (e.g. can you tell me a bit more about that?) and build (e.g. that’s interesting, do you notice other people in the community who influence your behaviour?) on the conversation. Rounds of practice conversations offered Community reporters the opportunity to refine their skills and approach until we were all comfortable with the task.
4. Final review and distribution of conversation materials (15 min)
The Community reporters had the opportunity to ask questions before being given information sheets, a link to the online consent form and a Dictaphone. Contact details for AP and the CEC were also provided, and AP ensured the Community reporters were competent in collecting stories and addressed any final questions.

### Anecdotal reflections

This CR approach generated important insights on local PA provision. For example, we discovered how small changes would expand the numbers of South-East Asian women using leisure provision and the importance of providing female deliverers of a similar cultural background to engage these women (e.g. by having only women lifeguards present at women only swimming sessions). Furthermore, the Community reporters revealed the importance of day-to-day social processes and how the essential role social networks play in validating involvement in PA (e.g. local parent groups organising postschool drop-off walking or running groups). Activating these social local influences will be essential when considering locally driven PA provision.

## Conclusion

In this article, we introduce and describe CR as an approach to gaining insight on local context from local residents. This may be useful for researchers, evaluators and practitioners working to understand local contexts and underserved groups. The CR approach offers an opportunity to work with community-based individuals to generate insights into local priorities and concerns. These issues can help address inequalities and should be considered by those who devise policies and strategies, and those working on delivering PA provision.
